# A dataset of cassava whitefly count images

**DOI:** 10.1016/j.dib.2022.107911

**Published:** 2022-02-03

**Authors:** Joyce Nakatumba-Nabende, Jeremy Francis Tusubira, Claire Babirye, Solomon Nsumba, Christopher Omongo Abu

**Affiliations:** a Department of Computer Science, Makerere University, Uganda; b Makerere University Artificial Intelligence Lab, Uganda; c Root Crops Research Programme, National Crops Resources Research Institute, Uganda

**Keywords:** Cassava, Whitefly, Whitefly count, CBSD

## Abstract

Whiteflies are insect vectors that affect a variety of plants such as tomatoes, cabbages, sweet potatoes, eggplants, and cassava. In Uganda, whiteflies are a major contributor to the spread of Cassava Brown Streak Disease (CBSD). By suckling on infected cassava plants, whiteflies can potentially transfer the Cassava Brown Streak Virus that causes CBSD to uninfected clean plants nearby when they migrate. When they attack the cassava plants in large numbers, whiteflies can also cause significant physical damage through suckling. This eventually can lead to leaf loss or plant death. Whiteflies also excrete “honeydew”, which harbors a fungus known as “sooty mold” that covers the leaves, limiting access to sunlight which in turn affects plant food production. As part of their work, the cassava breeders often conduct studies to assess the population of whiteflies in cassava fields through a manual process of visual inspection which can be arduous and time-consuming. This paper presents a cassava whitefly dataset that has been curated to enable researchers to build solutions for the automation of the count and detection of whiteflies. The dataset contains 3,000 images captured in a whitefly trial site in Uganda. It depicts different variations of whitefly infestation from low to high infestation. This data has already been used to provide a proof-of-concept solution for whitefly counting based on Machine Learning approaches.

## Specifications Table

**Table:** 

Subject	Computer Vision, Machine Learning, Agriculture
Specific subject area	Whiteflies on Cassava Leaves
Type of data	Raw image data
How data were acquired	13-megapixel smartphone cameras were used to capture the pictures of cassava infested whitefly leaves.
Data format	Images (JPEG) of cassava leaves infested with whiteflies. Annotations for the labeled whiteflies are stored as XML files. The data is organized in three categories based on the whitefly abundance on the images of the cassava leaves.
Parameters for data collection	Image data collected during cassava whitefly trial harvests from randomly selected plants.
Description of data collection	Images were collected in the field from cassava plants in Namulonge.
Data source location	Institution: National Crop Resources Research Institute (NaCRRI) Town: Namulonge Country: Uganda Location: Cassava whitefly trial fields at NaCRRI (00 32° N of the Equator and 320 37° E)
Data accessibility	Data is publicly available at Mendeley data. Repository name: Mendeley Data identification number: 10.17632/5g38399z9p.2 Direct URL to data: https://data.mendeley.com/datasets/5g38399z9p/2 The dataset citation is in Ref [1].
Related research article	Tusubira, J.F., Nsumba, S., Ninsiima, F., Akera, B., Acellam, G., Nakatumba, J., Mwebaze, E., Quinn, J. and Oyana, T., 2020. Improving In-field Cassava Whitefly Pest Surveillance with Machine Learning. In Proceedings of the IEEE/CVF Conference on Computer Vision and Pattern Recognition Workshops (pp. 68–69) [2]. DOI: 10.1109/CVPRW50498.2020.00042

## Value of the Data

• The whitefly image data can be used to train machine learning models to perform automated whitefly surveillance tasks by cassava breeders. The automation of whitefly detection as shown in [2] can help to improve the efficiency and accuracy of traditional whitefly counting methods described in [5]. The availability of the images also provides the opportunity to conduct evidence-based verification of whitefly count field surveys and to easily conduct new studies on collected data.
• The primary beneficiaries of the whitefly image data are researchers who can apply computing algorithms in the agriculture domain. By conducting tasks such as computer vision, the data can be used to develop models and systems for performing tasks like whitefly counting. Cassava breeders can utilize the developed systems to potentially improve the efficacy of whitefly studies and develop new standards and methods for performing the critical task of whitefly counting.
• This cassava whitefly image dataset provides an intersection between the computing and agricultural communities to collaborate towards building tools adaptable to the agriculture domain. The dataset can be used as a benchmark to evaluate the ability of machine learning object detection models to generalize to data from a different domain. This data can also be used to train weights that can be used in transfer learning for performing similar tasks.

## 1 Data Description

**Fig. 1:**
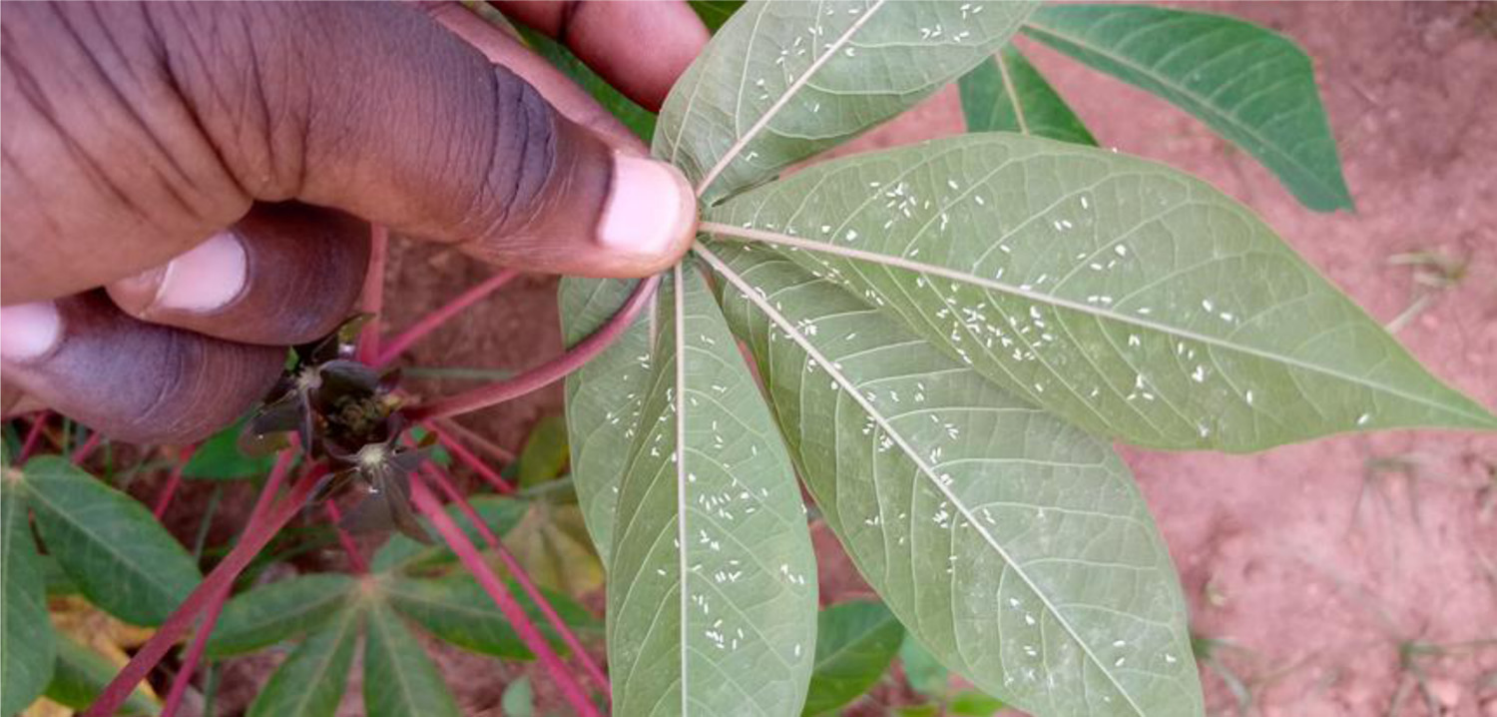
A sample image captured showing whiteflies on the backside of the leaves.

**Fig. 2:**
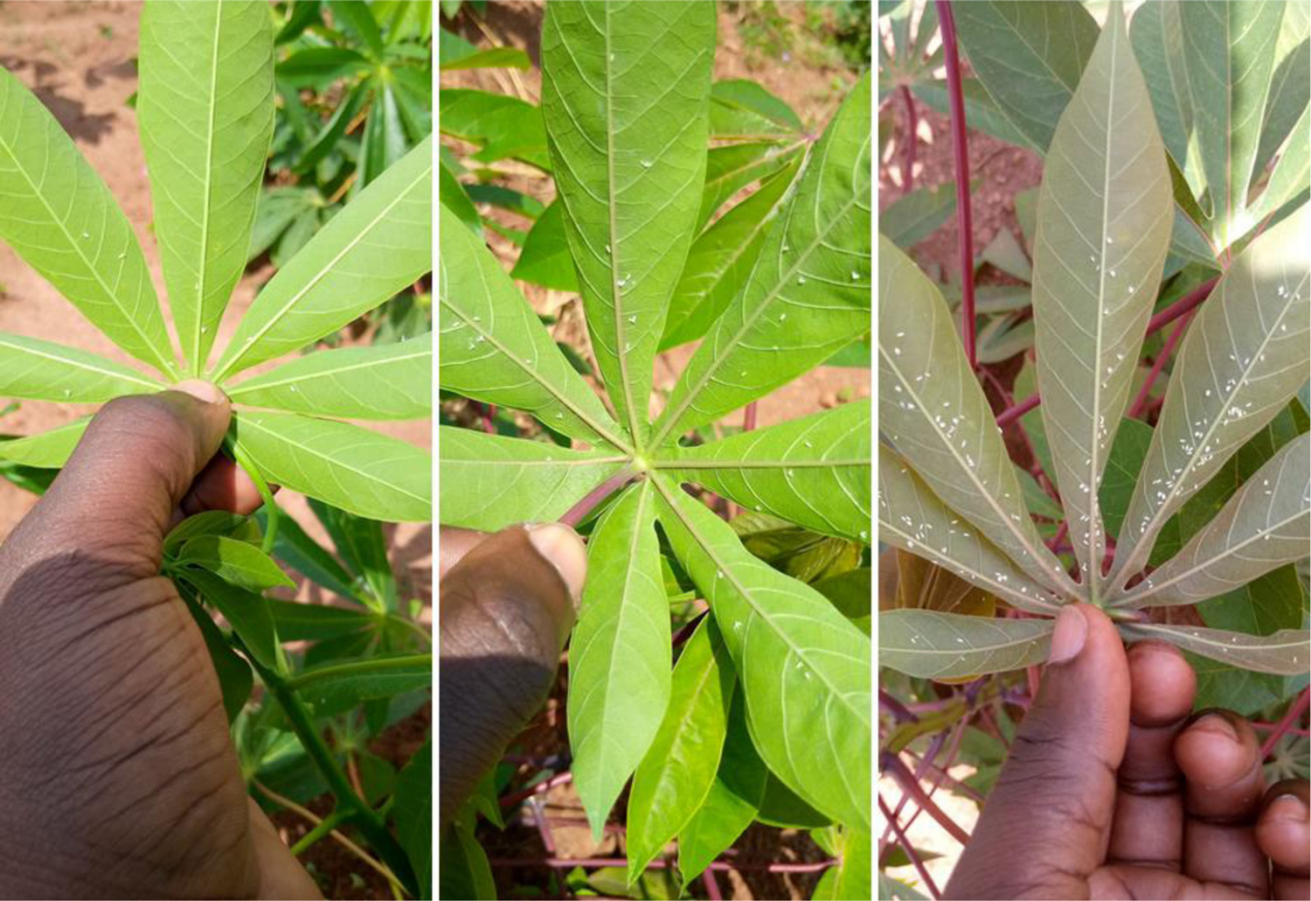
Sample images for each whitefly abundance category. The image on the left shows a cassava leaf with low abundance of whiteflies, the middle image shows a leaf with moderate abundance and the image on the right shows a cassava leaf with super abundance.

We present a dataset that contains images of whitefly-infested cassava leaves captured from cassava fields located at the National Crop Resources Research Institute (NaCRRI). The data contains images of adult whiteflies, which are one of the leading contributors to the spread of Cassava Brown Streak Disease (CBSD). The images were captured from the top open cassava leaves of randomly selected cassava plants. The technique used to capture the image data is suitable for monitoring the populations of adult whiteflies in the field [3]. The data is presented through two figures and one table. Fig. 1 shows an example of a sample leaf captured with the whiteflies. Fig. 2 shows images that depict different levels of population count of the whiteflies on the cassava leaves. Table 1 provides a description of the category of whitefly count abundance. The dataset contains 3000 images and their corresponding annotation files. This raw dataset is publicly available as a Mendeley repository [1]. The dataset presented is sufficient for training object detection models. This is based on the experiments conducted in [2] where a sample of 2000 images was sufficient to train a whitefly detection model with high precision.

The adult female whiteflies are found on the underside of the cassava leaves and the leaf stalks [8]. To capture the whitefly images, the cassava leaves were turned to reveal the underside of the leaves shown in Fig. 1. The whiteflies can be seen as tiny white specks on the infested leaves.

## 2 Experimental Design, Materials and Methods

### 2.1 Field data collection

Image cassava whitefly data was collected from Namulonge, the National Crops Resources Research Institute (NACRRI). Namulonge is located in the central region of Uganda in Wakiso District and is at 00 32″ N of the Equator and 320 37″ E. Namulonge has an average temperature of 20 C, Wind E at 2 km/h, and 90% Humidity. It is located 10 km north of Gayaza and 30 km North East of Kampala, the capital city of Uganda at an elevation of 1160 m above sea level. The image data was collected from cassava trial gardens set up for the purpose of studying whiteflies populations on cassava leaves, by the cassava breeders. The images were captured using a Tecno Spark 3 which is an android-based smartphone with a camera resolution of 13 megapixels. This produces JPEG images with a dimensions of 4000 × 1920 pixels or vice-versa depending on the orientation of the camera when the picture was taken as shown in Fig. 1. The cassava whitefly image data was collected from the NAROCASS 1 variety [9] which is a commercially grown cassava variety in Uganda. The cassava was at age three months after planting.

**Table 1:** Category of whitefly count abundance.

Category/Abundance	Whitefly count (WC)
Low	Less than 10
Moderate	Between 10 - 99
Super	Greater than 99

Traditional approaches estimate whitefly count by sampling five of the top most fully grown cassava leaves on the plant [5] and counting the number of fully grown visible whiteflies on each leaf. According to [6] and as shown in Table 1 whitefly count (WC) can be grouped into three levels of abundance;

1. Low abundance where the whitefly count is less than 10.
2. Moderate abundance where the whitefly count is between 10 and 100.
3. Super abundance where the whitefly count is greater than 100.

Fig. 2 shows sample cassava image files for each of the whitefly abundance categories described in Table 1.

### 2.2 Data preprocessing

**Fig. 3:**
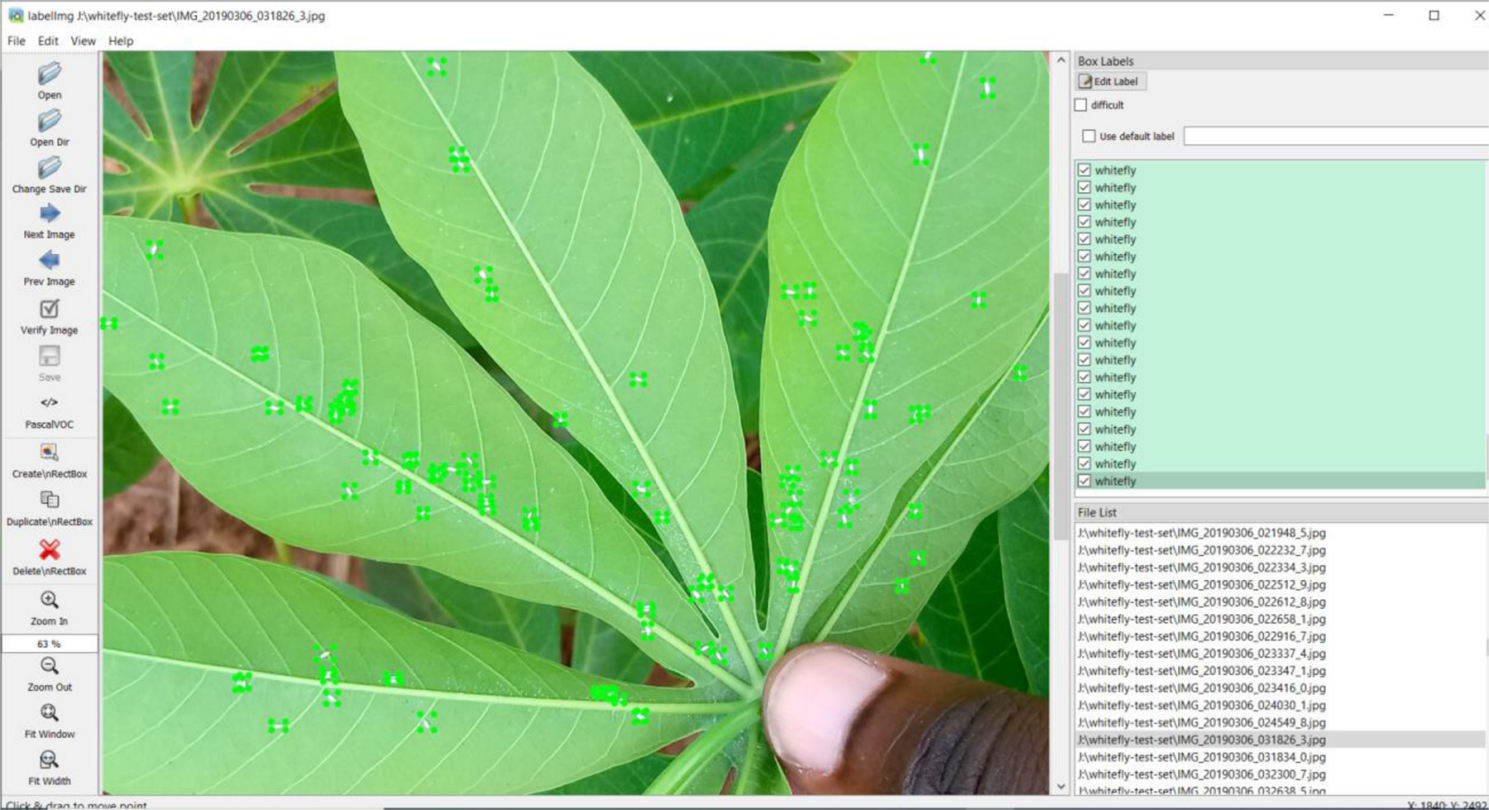
Image annotation using the LabelImg tool. Adapted from [2].

The data was labeled for the task of using computer vision techniques to identify and provide a count of the number of whiteflies in the images. Objects of interest were labeled by drawing bounding boxes around each whitefly and tagging each box with a corresponding text label which was “whitefly”. The bounding boxes were drawn using an open-source image annotation tool called LabelImg^1^ as shown in Fig. 3. The LabelImg tool stores the annotations in the PASCAL VOC (VisualObject Classes) [4] format which is a popular format for use in computer vision object detection tasks. Each annotation is an XML (Extensible Markup Language) [7] file that contains information about the positions of the whitefly objects in each image and the text label assigned to each object

Using the WC abundance definition in [6], the images in this dataset have been placed into three categories; images with a low whitefly abundance, images with a moderate whitefly abundance and images with a super abundance of whiteflies. The WC in each image was determined by counting the number of annotated whitefly objects in the corresponding annotation file.

## Ethics Statement

The work presented in this paper is original and has not been published or submitted for consideration anywhere else. All the required consent to collect and publish the data was gathered from the responsible parties and the data is available in the public domain.

## CRediT authorship contribution statement

**Joyce Nakatumba-Nabende:** Conceptualization, Writing – original draft. **Jeremy Francis Tusubira:** Data curation, Methodology, Writing – review & editing. **Claire Babirye:** Writing – review & editing. **Solomon Nsumba:** Writing – review & editing. **Christopher Omongo Abu:** Writing – review & editing.

## Declaration of Competing Interest

The authors declare that they have no known competing financial interests or personal relationships that could have appeared to influence the work reported in this paper.

## Footnotes

1 https://github.com/tzutalin/labelImg.
